# Quantum Magnetism of the Iron Core in Ferritin Proteins—A Re-Evaluation of the Giant-Spin Model

**DOI:** 10.3390/molecules29102254

**Published:** 2024-05-11

**Authors:** Wilfred R. Hagen

**Affiliations:** Department of Biotechnology, Delft University of Technology, Building 58, Van der Maasweg 9, 2629 HZ Delft, The Netherlands; w.r.hagen@tudelft.nl

**Keywords:** ferritin, core, EPR, spin Hamiltonian, zero-field interaction, higher-order terms, giant spin, metal-ion clusters, single-molecule magnets, superparamagnetism

## Abstract

The electron–electron, or zero-field interaction (ZFI) in the electron paramagnetic resonance (EPR) of high-spin transition ions in metalloproteins and coordination complexes, is commonly described by a simple spin Hamiltonian that is second-order in the spin *S*: $H=D[S_{z}^{2}-S\left(S+1\right)/3+E(S_{x}^{2}-S_{y}^{2})$. Symmetry considerations, however, allow for fourth-order terms when *S* ≥ 2. In metalloprotein EPR studies, these terms have rarely been explored. Metal ions can cluster via non-metal bridges, as, for example, in iron-sulfur clusters, in which exchange interaction can result in higher system spin, and this would allow for sixth- and higher-order ZFI terms. For metalloproteins, these have thus far been completely ignored. Single-molecule magnets (SMMs) are multi-metal ion high spin complexes, in which the ZFI usually has a negative sign, thus affording a ground state level pair with maximal spin quantum number *m*_S_ = ±*S*, giving rise to unusual magnetic properties at low temperatures. The description of EPR from SMMs is commonly cast in terms of the ‘giant-spin model’, which assumes a magnetically isolated system spin, and in which fourth-order, and recently, even sixth-order ZFI terms have been found to be required. A special version of the giant-spin model, adopted for scaling-up to system spins of order *S* ≈ 10^3^–10^4^, has been applied to the ubiquitous iron-storage protein ferritin, which has an internal core containing Fe^3+^ ions whose individual high spins couple in a way to create a superparamagnet at ambient temperature with very high system spin reminiscent to that of ferromagnetic nanoparticles. This scaled giant-spin model is critically evaluated; limitations and future possibilities are explicitly formulated.

## 1. Introduction

**Figure 1:**
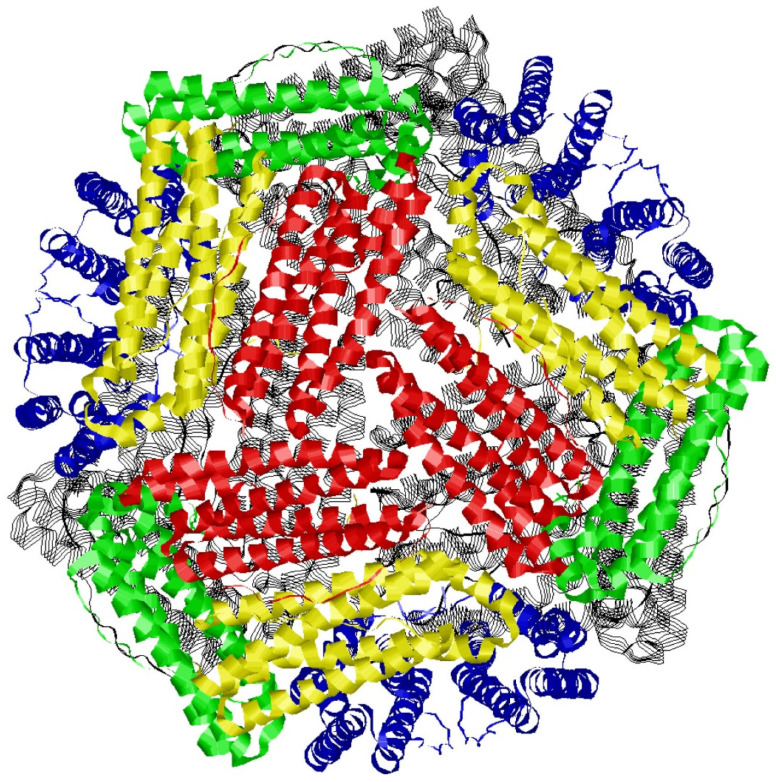
Crystallographic model of the ferritin from *Pyrococcus furiosus*. The structure (2JD7.pdb [11]), obtained from an Fe-soaked crystal, consists of 24 identical subunits each of mass 20.3 kD and each with a five-helical bundle fold. Twelve subunits have been colored in groups of three to emphasize 3-fold symmetry (e.g., three red subunits) and 4-fold symmetry (where red, green, yellow, and blue subunits meet). The other 12, in the back, are all in black. The most conspicuous aspect of the figure is the complete absence of an internal core structure, reflecting the fact that atomic-structure resolution has never been obtained for any core in any ferritin.

The ubiquitous protein ferritin is made up of 24 relatively small subunits of 20 kDa, which form a quasi-spherical shell of 8 nm inner diameter (Figure 1), whose biological function is to store iron inside its cavity until metabolism requires its release [1,2,3,4]. Enzymatic activity of ferritin encompasses uptake of Fe^2+^, oxidation to Fe^3+^ in ferroxidase active sites, and transfer of Fe^3+^ to the cavity for mineralization [5,6,7]. The protein has been intensively studied from a range of eukaryotic, archaeal, and bacterial species, stimulated by its exceptional thermal stability, ease of heterological expression and purification, and tendency to readily crystallize [8]. Thus, multiple decades (since 1937 [9]) of intense structural and mechanistic studies have resulted in significant understanding of ferritins’ molecular biology. By contrast, our knowledge on ferritins’ core structure, formation, and dissolution, is incomplete and controversial. It is, for example, common in ferritin literature to refer to the core structure as ‘ferrihydrite-like’, wherein the degree of likeness is never specified. It also does not help that ferrihydrite itself is a complex system, whose ‘idealized structure’ for domains of 2–6 nm has been reported to have the chemical formula Fe_10_O_14_(OH)_2_, however, with the caveats that individual domains are subject to disorder, surface relaxation, internal strain, and defects [10]. Perhaps the key question in this type of comparison is whether a single-phase model, as developed for ferrihydrite grains, is applicable to ferritin cores, where the composition of the latter is known to depend on many intrinsic and environmental parameters, such as the biological source; subunit composition; degree of loading (that is, how many iron ions per core); presence of solutes, in particular phosphate; temperature of formation; rate of formation; nature of oxidant; competition with non-biological background Fe^2+^ oxidation; etc. Clearly, core heterogeneity can be expected to be a formidable complicating factor in any attempt to understand the core’s chemistry.

In spite of this intrinsic risk factor, ‘the’ magnetism of ferritin cores has been a popular object of research from early on [12] up to this day [13]. For many years, these studies were interpreted in the framework of classical magnetism with its concepts of (anti)ferromagnetism and superparamagnetism (for EPR studies, see, e.g., [14,15,16,17]). However, more recently, a putative identification of ferritin cores as positioned at the borderline between classical magnetic and quantum magnetic systems has led to attempts [13,18,19] to describe cores by means of spin Hamiltonians of a special type, known as the ‘giant-spin model (GSM)’ [20,21]. GSM was initially developed for single-molecule magnets of much smaller size than ferritins, such as the prototypical ‘Mn_12_ac’ molecule [Mn_12_O_12_(CH_3_COO)_16_(H_2_O)_4_]·2CH_3_COOH·4H_2_O [22] with a ground state *S* = 10 [23]. In these studies, ‘giant spin’ (in early work ‘large spin’ [20]) was loosely defined as *S* >> 1 [21], and the model may already take effect for *S* = 4 [24]. Of a different order, the ground-state spin of presumed single-domain core in ferritin has been taken to be as high as *S* = 5000 [19]. Such a truly giant spin, in combination with documented core heterogeneity, leads to interpretational ambiguities. The resulting limitations, both of physics and biochemical nature, are the subject of this perspective.

## 2. Zero-Field Interaction in Mononuclear Complexes

The paramagnetism of transition ion complexes is generally described with a spin Hamiltonian, that is, an effective Hamiltonian whose validity is limited to the magnetic ground manifold of states [25]. This approach has several goals. A first and foremost one is to provide a straightforward means to tabulate spectroscopic data where knowledge on electronic structure may (yet) be limited or even absent. For example, EPR spectra of mononuclear high-spin iron(III) compounds are frequently described with the well-known spin Hamiltonian:

(1) $$H=D\left[S_{z}^{2}-S\left(S+1\right)/3\right]+E\left(S_{x}^{2}-S_{y}^{2}\right)+\beta B\cdot g\cdot S$$

which means that spectra can be catalogued by means of the few parameters, *D*, *E*, *g*_x_, *g*_x_, and *g*_z_. In fact, realizing that quenching of orbital angular momentum in the half-filled shell of 3d^5^ affords *g*_x_ ≈ *g*_y_ ≈ *g*_z_ ≈ 2.00, and with the frequent observation that *D* >> *gβB* at conventional EPR microwave frequencies, one may find that apparently complicated spectra, comprised of several inter-doublet transitions, each one with its own set of effective *g* values, can be listed by means of one single parameter only, the rhombicity *η* = *E*/*D*, in charts of effective *g* values versus rhombicity called rhombograms [26]. In many practical cases, it may be necessary to add a second parameter in the form of a standard deviation, *σ*_η_, for a statistical distribution in rhombicities, or in *E*-values [27]. An obvious second goal is to learn about electronic and geometric molecular structures by modelling them in a format that allows for linking of their variables to the numerical values of the spin Hamiltonian parameters. A relatively simple example would be low-spin Fe^3+^ in hemes and hemoproteins, whose EPR is described by the electronic Zeeman interaction only, that is, the last term in Equation (1), where the values of *g*_x_, *g*_y_, and *g*_z_ can be related to the splittings between the *d*_xy_, *d*_xz_, and *d*_yz_ positive hole levels in terms of axial and rhombic distortion from quasi-octahedral symmetry [28]. Even when the linkage between spin-Hamiltonian parameters and actual structure may be hard to make, for example, for lack of structural information, or because EPR in distributed systems is insufficiently informative, the spin Hamiltonian approach may have a third goal, namely as a predictor of possible powder-pattern EPR line shapes. This application is of particular importance in biomolecular EPR analysis of multi-center metalloproteins with the objectives of establishing stoichiometries [29], and of identifying spectral components with particular centers to allow for their individual monitoring, e.g., in redox titrations (e.g., [30]).

A zero-field spin Hamiltonian that gives a complete description of multipole fine structure from the crystal-field potential is given by the series of terms:

(2) $$H=\sum_{k,q}B_{k}^{q}O_{k}^{q}$$

in which the *B*’s are the Stevens constants of the spin operators *O* [25,31]. The values of *k* and *q* are determined (that is, limited) by the symmetry of the crystal field, whereby *k* is furthermore limited by the spin of the system, such that *k* ≤ *S*, and by time-reversal invariance of H, such that *k* is even. In biomolecular EPR this causes a fundamental as well as a practical problem: metal sites in proteins formally do not possess any symmetry at all, if only because amino acids are all levorotatory; however, spectral effects of symmetry lower than orthorhombic (that is, monoclinic or triclinic) are very hard to discern in the absence of single-crystal EPR data. Therefore, attempts to apply low-symmetry spin Hamiltonians to biomolecules are rare and typically do not involve Equation (2), but rather a tensor non-colinearity between the *g*-matrix and a metal hyperfine tensor, as with low-spin Co^2+^ in vitamin B_12_ [32] or with Cu^2+^ bound to transferrin [33]. For zero-field interactions a description based on orthorhombic, or higher, symmetry is generally, and tacitly assumed to be sufficient. For example, the spin Hamiltonian in Equation (1) is one that applies to orthorhombic symmetry (that is, point group *D*_2h_, *D*_2_, or *C*_2v_ [34]. However, it is important in the present framework to realize that Equation (1) is not complete. The complete orthorhombic zero-field Hamiltonian for 2 ≤ *S* ≤ 5/2 is:

(3) $$H=B_{2}^{0}O_{2}^{0}+B_{2}^{2}O_{2}^{2}+B_{4}^{0}O_{4}^{0}+B_{4}^{2}O_{4}^{2}+B_{4}^{4}O_{4}^{4}$$

in which the first two terms are equivalent to the first two terms in Equation (1) by virtue of:

(4) $$B_{2}^{0}\equiv D/3;\;B_{2}^{2}\equiv E$$

and the last three terms are simply missing, as seen from the expressions of the spin operators in terms of angular momentum *S* [25,31]:

(5a) $$O_{2}^{0}=3S_{z}^{2}-S(S+1)$$

(5b) $$O_{2}^{2}=1/2(S_{+}^{2}+S_{-}^{2})$$

(5c) $$O_{4}^{0}=35S_{z}^{4}-\left[30S\left(S+1\right)-25\right]S_{z}^{2}-6S\left(S+1\right)+3S^{2}{\left(S+1\right)}^{2}$$

(5d) $$O_{4}^{2}=1/4\{\left[7S_{z}^{2}-S\left(S+1\right)-5\right]\left(S_{+}^{2}+S_{-}^{2}\right)+\left(S_{+}^{2}+S_{-}^{2}\right)\left[7S_{z}^{2}-S\left(S+1\right)-5\right]\}$$

(5e) $$O_{4}^{4}=1/2(S_{+}^{4}+S_{-}^{4})$$

**Figure 2:**
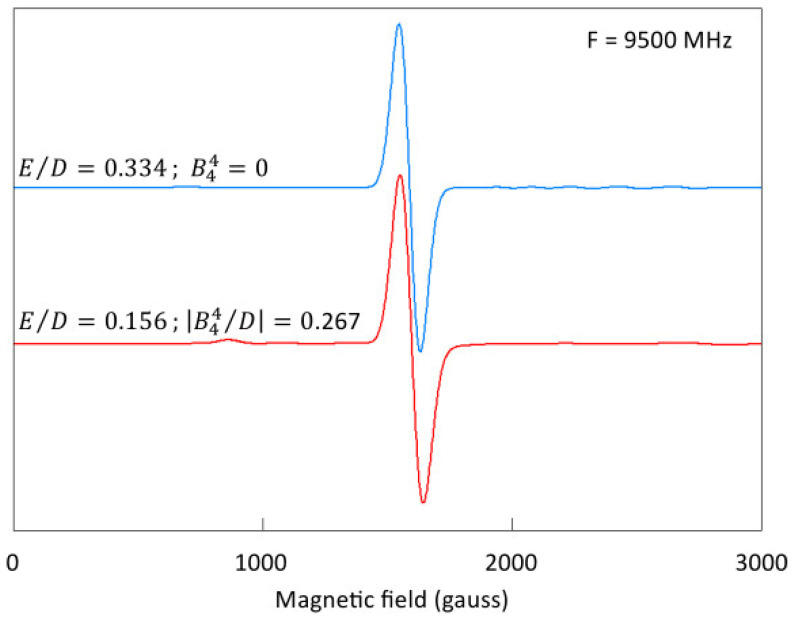
Very similar simulations of a *g* = 4.3 signal based on very different spin Hamiltonians. The ubiquitous *g* = 4.3 signal is generally considered to stem from maximally rhombic high-spin Fe^3+^ with *E*/*D* = 1/3. The blue trace was generated with the spin Hamiltonian in Equation (1) with *D* = −0.9 cm^−1^ and *E* = −0.3 cm^−1^. The red trace is based on Equation (1) extended with the fourth-order term in Equation (5e) using *D* = −0.9 cm^−1^, *E* = −0.14 cm^−1^, and *B*^4^_4_ = 0.24 cm^−1^. For both traces, *g*_iso_ = 2.00, and the temperature *T* = 15 K. The line shape is Gaussian with FWHM = 100 gauss.

The lack of the terms in Equation (5c–e) from Equation (1) now poses a different problem in the EPR analysis of distributed systems (such as frozen solutions of metalloproteins), which is perhaps more consequential than ignoring the complete lack of symmetry in macromolecular biosystems. For some systems, for example the high-spin Fe^3^ in ferrimyoglobin [35], the symmetry may be assumed to be approximately axial (tetragonal), and this would eliminate Equation (5b), and thus the second term in Equation (1), as well as Equation (5c). One may even envision a system to have cubic symmetry by approximation, for example, Mn(II) aquo in protein binding studies [36]. However, higher than cubic, that is, spherical, symmetry would never be a realistic assumption in biology, and, therefore, the elimination of Equation (5a,e) is never formally allowed. Still, these two terms of fourth order in *S*, which would, in fact, combine into one for cubic symmetry [25] as:

(6) $$H=B_{4}(O_{4}^{0}+{5O}_{4}^{4})$$

are missing in Equation (1). Nevertheless, although their inclusion in biological EPR analysis is rare, the few available examples, Fe^3+^ *S* = 5/2 in some siderophores [37] or an *S* = 2 system in O_2_-activated cytochrome oxidase [38], suggest that quartic terms should not be ignored. This point is illustrated in Figure 2 on a model simulation of the well-known *g* = 4.3 EPR spectrum from maximally rhombic high-spin Fe^3+^ which can equally well be obtained on the basis of intermediate rhombicity plus a finite *B*^4^_4_ term.

In what is obviously a circular argument, many authors have justified the use of Equation (1) by simply stating that it is ‘the usual Hamiltonian’. Occasionally, in the literature on non-biological complexes, it is contended that Equation (2) (and its higher-order equivalents for *S* > 5/2) would behave as a series expansion, that is, a (hopefully) converging series approximation (e.g., [39]). In other words, higher-order terms should be seen as perturbations of lower-order ones: the higher their order is, the smaller their numerical contribution to the zero-field splittings is, and the more justified it would be to ignore them. This interpretation has two issues: (i) there is no objective convergence test other than a full-blown analysis involving all symmetry-allowed terms, and (ii) even limiting higher-order coefficients to values that are several orders of magnitude less than their lower-order counterparts does not necessarily minimize their contribution because the numerical values of the matrix elements of the operators *O* tend to rapidly increase with the order of the operator. This is readily appreciated by comparing the tables of matrix elements of *O*’s, e.g., given in Appendix B, Table 17 of [25]; it is also illustrated in an example worked out in Section 4, below.

## 3. Zero-Field Interaction in Polynuclear Complexes

Does the picture, outlined above, change when two, or more paramagnetic ions are grouped in a single molecule at relatively short distances? For example, in iron-sulfur proteins and their chemical models, iron ions, possibly in different oxidation states, but always in high-spin configurations, are typically μ3-S bridged into clusters, with their coordination completed by external thiolate ligands. The individual metal ions are subject to strong magnetic coupling, which is a combination of superexchange (tending to localize valencies) and double exchange (tending to delocalize valencies). The result is a spin ladder with energy separations of the order of 100 wavenumbers ([40] and references therein), that is, well exceeding the X-band microwave energy of 0.3 cm^−1^, so at helium temperatures, the ground state is an isolated magnetic manifold with a characteristic system spin. The only conspicuous difference with the mononuclear d-systems is the possibility of *S* > 5/2. Indeed, clusters in proteins have been reported with system spin *S* = 3 [41,42], *S* = 7/2 [43,44], *S* = 4 [41,45], *S* = 9/2 [46,47,48], and *S* = 11/2 [49] In principle, these spins can be rationalized in an Aufbau approach, in which intermediate spins are assigned to dimers of iron ions, which subsequently are combined into a complete cluster ([26,50] and references therein). In these systems, ions cannot be separated to study their individual magnetic properties, but qualitative comparison can be made with clusters of lower nuclearity and with mononuclear sites. From an EPR spectroscopic perspective, the approach described above for single ions applies equally well to these clusters with the qualifier that Equation (3) should be extended with *O*_6_ terms for *S* ≥ 7/2 furthermore with *O*_8_ terms for *S* ≥ 9/2, etc. In practice, analysis has invariably been limited to application of the incomplete Hamiltonian in Equation (1) with disregard of any ZFI term with *k* > 2.

Research on a new class of synthetic polynuclear transition ion complexes, generally known as single-molecule magnets (SMMs), started off in the 1990s and continues to bear fruit today. Typically, the magnetism of these molecules, with order-of-magnitude sizes of 10^2^ atoms, differs from that of, e.g., iron-sulfur proteins not in any fundamental sense but in practical aspects. Their limited size affords high concentrations in powders and in single-domain crystals. On average, the strength, *J*, of intramolecular superexchange coupling (here, through μ_2_-O and/or μ_3_-O bridges) between metal ions appears to be somewhat weaker than in iron-sulfur clusters, which implies a reduced energy separation between the ground state and excited spin multiplets. Entangling interference between these states at laboratory magnetic fields became evident due to the incidentally concurrent development of the fields of SMMs and of high-field/high-frequency (hf) EPR spectroscopy.

Magnetically, the simplest giant spins occur in homopolynuclear SMMs with the metal ions in a single oxidation state, e.g., ‘Ni_4_’ [Ni(hmp)(ROH)Cl]_4_ (hmp is 2-hydroxymethylpyridine; R is, e.g., CH_3_) with four Ni(II) *S* = 1 spins that simply add up by parallel exchange to a total spin *S* = 4 [51,52]. Equally, in ‘Ni_12_’ [Ni_12_(chp)_12_(O_2_CMe)_12_(H_2_O)_6_(THF)_6_] (chp is 6-chloro-2-pyridonate), twelve *S* = 1 spins add up to *S* = 12 [53]. Somewhat more involved are systems where different ions may have different spins that may lead to ‘incomplete antiparallel compensation’, as, e.g., in the already mentioned ‘Mn_12_’ compound with eight Mn(III) *S* = 2 ions and four Mn(IV) *S* = 3/2 ions affording a system spin of 8 × 2 − 4 × 3/2 = 10 [54,55]. More involved coupling schemes also arise, e.g., in the all ferric ‘Fe_8_’ [Fe_8_O_2_(OH)_12_(tacn)_6_]^8+^ (tacn is triazacyclononate) with eight Fe(III) *S* = 5/2 ions, where the system spin is modelled as coming from four Fe spins that straightforwardly couple parallel, but the other four spins couple in a complex manner into a subspin *S* = 0, so that the total spin is 4 × 5/2 + 0 = 10 [56,57]. More involved systems have also been found, such as ‘Mn(IV)Mn(III)_3_’ *S* = 9/2 [58,59], ‘V(III)_4_’ *S* = 3, ‘Cr(III)_4_’ *S* = 1, ‘Mn(III)_4_’ *S* = 3 [60], ‘Mn_30_’ *S* = 7 [61], ‘Mn_18_’ *S* = 13 [62], ‘Mn_25_’ *S* = 51/2 [63], ‘Fe_6_’ *S* = 19/2 [64,65], and ‘Mn_3_’ *S* = 6 [66]. The zero-field interaction in most if not all of these complexes appears to be dominated by an axial term (Equations (1) and (4)), with a negative *D* value resulting in a magnetic ground multiplet in which the lowest level pair is the *m*_S_ = ±*S*_max_. In (close to) zero magnetic field, and at sufficiently low temperatures, when these are the only significantly populated levels, the thermal barrier between the true ground state *m*_S_ = −*S*_max_ and the first excited state *m*_S_ = +*S*_max_ makes the system an SMM [67]. Occurrence of tunnelling relaxation between them furthermore makes the molecule a potential switching element for quantum computing [68]. However, a third aspect of the molecular magnetism of these compounds has a more relevant bearing on the present perspective: in several reports it is claimed that a quantitative description of their EPR calls for inclusion of higher order zero-field terms in the spin Hamiltonian [24,51,55,57,58,65,66].

For reference in what follows, the sixth-order zero-field Hamiltonian in orthorhombic symmetry is:

(7) $$H=B_{6}^{0}O_{6}^{0}+B_{6}^{2}O_{6}^{2}+B_{6}^{4}O_{6}^{4}+B_{6}^{6}O_{6}^{6}$$

and these are the equivalent sixth-order terms in *S* [25,31]:

(8a) $$O_{6}^{0}=231S_{z}^{6}+\left[-315S\left(S+1\right)+735\right]S_{z}^{4}+[105S^{2}{\left(S+1\right)}^{2}-525S\left(S+1\right)+294]S_{z}^{2}\;-5S^{3}{\left(S+1\right)}^{3}+40S^{2}{\left(S+1\right)}^{2}-60S(S+1)$$

(8b) $$\begin{array}{r} O_{6}^{2}=1/4\{\left[33S_{z}^{4}-18S_{z}^{2}S\left(S+1\right)-123S_{z}^{2}+S^{2}{\left(S+1\right)}^{2}+10S\left(S+1\right)+102\right]\left(S_{+}^{2}+S_{-}^{2}\right)\\ +\left(S_{+}^{2}+S_{-}^{2}\right)[33S_{z}^{4}-18S_{z}^{2}S\left(S+1\right)-123S_{z}^{2}+S^{2}{\left(S+1\right)}^{2}+10S\left(S+1\right)+102]\} \end{array}$$

(8c) $$\begin{array}{r} O_{6}^{4}=1/4\{[11S_{z}^{2}-S\left(S+1\right)-38]\left(S_{+}^{4}+S_{-}^{4}\right)\\ +\left(S_{+}^{4}+S_{-}^{4}\right)[11S_{z}^{2}-S\left(S+1\right)-38]\} \end{array}$$

(8d) $$O_{6}^{6}=1/2(S_{+}^{6}+S_{-}^{6})$$

Equivalent to the *S*^4^ expression in Equation (6), even in cubic symmetry, a single sixth-order term is allowed in the form of a combination of Equation (8a,d) [25]:

(9) $$H=B_{6}(O_{6}^{0}-21O_{6}^{6})$$

Originally, over a decade, magnetic susceptibility and EPR data were interpreted in terms of a minimalistic Hamiltonian with only a *D*-term (*B*^0^_2_) describing the zero-field interaction [23,53,54,59,60,62,63,67,69], with occasional inclusion of a rhombic *E*-term (*B*^2^_2_) [56,61]. Barra et al. were the first to notice that the cubic fourth-order terms, Equation (5c,e), had to be invoked for a reasonable description of hf EPR data from the canonical Mn_12_ac molecule. They also noted that the Equation (5e) term plays a crucial role in the mechanism of quantum tunneling [55]. Shortly afterward, interpretation of inelastic neutron scattering of Fe_8_ was found to require the complete orthorhombic zero-field Hamiltonian of Equation (3) [57]. A single *B*^0^_4_-term, Equation (5c), was also included in descriptions of several Mn^IV^Mn^iii^_3_ [58] and Ni_4_ clusters [51]. More recently, sixth-order terms were included in fits of hf EPR from an Mn_3_ complex with trigonal symmetry [66].

Wilson et al. initiated a discussion on the physical meaning, or the lack thereof, of higher-order terms in the spin Hamiltonian of SMMs [24]. They studied the hf EPR of a Ni_4_ complex, and their line of reasoning was as follows: the individual Ni ions all have *S* = 1; therefore, individual fourth-order terms are not allowed; therefore, any such term in the giant-spin Hamiltonian of the cluster must in some way reflect the exchange interactions between the ions, which in turn, by virtue of their limited magnitude determine coupling to higher lying states, or *S* mixing. This then leads to the proposal that the parameters in the giant-spin Hamiltonian should be linked to those in the many-spin Hamiltonian (MSH), that is, the spin Hamiltonian of all individual metal ions plus their mutual interactions. Eventually, this leads to a somewhat dichotomous conclusion: on the one hand, “*B*^0^_4_ is nothing more than an adjustable parameter in an effective model”; on the other hand, the results “clearly show that *J* can be estimated on the basis of the ZFS within the lowest lying multiplet” [24]. The following questions can be raised. (1) The four Ni ions form an approximate cube with approximate *S*_4_ symmetry [51], which means that symmetry allows three fourth-order and three sixth-order ZFI terms (Table 4-2 in [34]). Where are these terms in the description of the Ni_4_ EPR? (2) What if the metal ions (in other complexes) have individual spins *S* ≥ 2 and therefore have individual fourth-order terms? (3) What if the exchange coupling between metal ions is so strong that the ground spin state is thermally well isolated? Are fourth-order and higher terms then symmetry-allowed but practically absent? Several authors have worked out theoretical alternative approaches to deal with perceived shortcomings of the giant-spin approximation for SMMs [70,71,72]. The discussion is still very much ongoing.

## 4. Variants of the Giant-Spin Model for Truly Giant Spins

Fittipaldi et al. have proposed to extend applicability of the giant-spin model to nanoparticles, in particular to the ca 8 nm ferrihydride-like core of ferritin proteins [18,19]. The first problem that arises is the spin of the system: their estimate for fully loaded protein from the hyperthermophilic archaeon *Pyrococcus furiosus*, from susceptibility measurements, is *S* ≈ 5000 based on the relation:

(10) S=μ/gβ

in which *S* is the system spin, *μ* is its effective magnetic moment, *g* is the isotropic g value, and *β* is the Bohr magneton. This spin is over two orders of magnitude greater than the typical giant spins found for SMMs. Diagonalizing the energy matrix for an *S* = 5000 system is well beyond the capacity of standard computers both in terms of RAM usage and of CPU time. Their approach is to scale down to an effective spin *S*_eq_ (where the subscript is not explained, but presumably stands for ‘equivalent’), defined as:

(11) $$S_{eq}=S/n$$

where *S* is the real spin (here: 5000), and *n* is a positive integer. The proposal also included the statement that the zero-field parameter *D* (cf Equations (1) and (4)) in the spin Hamiltonian can be deduced from the relation:

(12) $$D=\gamma B_{a}/2S$$

in which *γ* is the electron gyromagnetic ratio, and *B*_a_ is the anisotropy field in the classical (that is, non-quantum) description of nanoparticle magnetism [19]. With Equation (12) and *B*_a_ = 1400 gauss (see [18,73] for its determination), they calculate a value of *D* = −1.2 × 10^−5^ cm^−1^. This number can be scaled up via the relation:

(13) $$D_{eff}=nD$$

which, for *n* = 500, affords the parameters *S*_eq_ = 10 and *D*_eff_ = −6 × 10^−3^ cm^−1^ to be used in simulations of EPR spectra. For temperature-dependent data, it is then also required to ‘simulate’ an experimental temperature as:

(14) $$T_{eff}=T/n$$

The above procedure has also been recently adopted by Bossoni et al. in an analysis of the magnetism and EPR of the core in ferritin isolated from human liver [13]. I add the following caveats: (i) the value of *D* is extremely small; even for the reduced spin *S*_eq_ = 10 the corresponding *D*-value is some two orders of magnitude smaller than typical values for SMMs or for iron-sulfur clusters. The physicochemical significance of this is unclear; comments are lacking in the relevant literature [13,18,19]. (ii) the classical model in which the factor *B*_a_ figures, assumes randomly oriented particles of variable size, however, with each particle being a single-domain magnet with uniaxial anisotropy [73]. It is not clear whether ferritin cores are single-domain magnets; it is far from obvious that they will have uniaxial symmetry. (iii) More generally, there is no reason why description of the zero-field interaction in a ferritin core should be limited to a single parameter (*B*^0^_2_) only.

**Figure 3:**
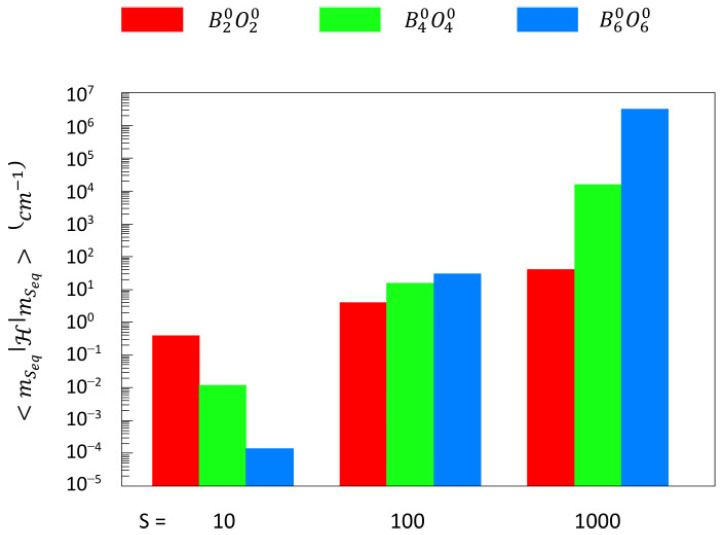
Non-scalability of axial higher-order ZFI terms for giant spins. Based on a reported value of *D* = −1.2 × 10^−5^ cm^−1^ with a spin of *S* = 5000 for the core of *Pyrococcus furiosus* ferritin [19], values of *B*^0^_2_ = −2 × 10^−3^, −2 × 10^−4^, and −2 × 10^−5^ cm^−1^ are deduced with Equation (13) for *S*_eq_ = 10, 100, 1000. Values for *B*^0^_4_ and *B*^0^_6_ are then taken to be less than *B*^0^_2_ by a factor of 10^4^ and 10^8^, respectively. The plotted number is the magnitude of the largest diagonal element in the energy matrix: $<m_{S_{eq}}\left|H\right|m_{S_{eq}}>$ for $H=B_{k}^{0}O_{k}^{0}$ with *k* = 2, 4, 6, respectively.

Experimental EPR data, and therefore spin Hamiltonian parameters, are not available for individual iron ions in ferritin cores. A surjection of individual-spin Hamiltonian parameters onto the giant-spin Hamiltonian is not possible. The only thing we have is the giant spin. Thus, there is no formal reason to limit the zero-field parameters to a *D*-value only. Making the common assumption of orthorhombic symmetry in biological EPR, all the terms in Equations (5) and (8) are allowed. In fact, a very large number of higher-than sixth-order terms is allowed when, e.g., *k* ≤ 5000 in Equation (2). How can we deal with these many terms? Can we order them in terms of importance, that is, magnitude? The answer is no. Even if the value of ZFI parameters would decrease by several orders of magnitude for each step-by-two increase in *k*, the mere size of the spin can easily compensate and make higher-order terms in *k* at least comparable to the lowest-order ones. I illustrate this effect for the axial terms in *O*^0^_2_, *O*^0^_4_, and *O*^0^_6_ in Figure 3, based on a *D* = −1.2 × 10^−5^ cm^−1^ as reported for *S* = 5000 [19], with a four orders-of-magnitude decrease for each subsequent *k*, that is, *B*^0^_4_ = 0.0001 × *B*^0^_2_ and *B*^0^_6_ = 0.0001 × *B*^0^_4_, where the first scaling corresponds to numbers reported for SMMs, quoted above, and the last scaling is a conservative estimate (cf [65]).

The fourth- and sixth-order terms can be seen to be reduced in size compared to the second-order term for *S* = 10; however, for *S* = 100, the three terms have become of comparable magnitude, and for *S* = 1000, each step in *k* leads to a large increase in the magnitude of *B*^0^_k_*O*^0^_k_. Clearly, a further increase in *k* will cause the axial zero-field interaction to eventually diverge versus infinity, which is in contrast with experimental observation (see below).

## 5. Structure and Crystallinity of the Core

A body of literature spanning seven decades is available on structural properties of the ferritin core, in particular on its shape, possible substructures, and crystallinity versus amorphousness. The list starts with a 1954 paper by Farrant [74]; the next 40 years have been comprehensively reviewed by Massover, with the following main conclusions: (i) the core exhibits substructure; (ii) the primary unit of substructure is a crystallite; (iii) the number of crystallites per ferritin is highly variable from one crystal to many crystallites that make up a single core; and (iv) multiple crystallites in a core do not have mutually ordered positions [75]. The large majority of cited studies have been limited to horse spleen ferritin.

The following three decades, until today, witnessed a continuing modulating discussion on these conclusions: the core would be comprised of one single phase [76], two phases [77,78], three phases [79], two phases arranged in up to eight subunits [80], three phases [81], back to two phases [82], and to a single phase [83]. Note that, again, the conclusions apply to mammalian ferritins. Less frequent studies on the core in ferritin of bacterial or archaeal origin were also mutually inconsistent, claiming two phases depending on loading degree [84], or essentially a single phase [82]. In the latter study, a breakdown of magnetic ordering was observed in microbial ferritins in the presence of phosphate, while, contrarily, a recent study reported a change from irregularly shaped to spherical shaped core for human H-chain ferritin [85]. Although the main conclusion must be that, after 70 years of research, a consensus view on core morphology and crystallinity remains something to wish for, it is also clear that the majority of studies suggest the core to be multi-phasic, and, therefore, that assigning a single spin to ferritin cores would be an unfounded assumption.

## 6. Biochemical Problems Related to Core Formation

The ferritin literature at large is pervaded with the notion that a single ferritin molecule can store up to some 4500 ferric ions. I have recently developed the view that this claim is unjustified and that it is a consequence of massive citation distortion. I have also proposed that the actual maximum loading number should be taken to be less than circa 3000 Fe [86]. In the work of Fittipaldi et al., in which the giant-spin model is applied to the core of *Pyrococcus furiosus* ferritin (PfF), an in vitro loading factor of apoprotein was claimed of 4500 Fe with subsequent analytical determination of 4050 Fe atoms/protein [18], or to about 4000 Fe atoms/protein [19]. These studies make no mention of our original work on the isolation, heterogeneous expression, and loading experiments of PfF, in which we reported a maximum loading of 2700 Fe [87]. In a later study, we found a maximum loading of 2500 Fe per PfF [88].

What does it mean to load a protein molecule with 4500 Fe, when it can actually only take up 2500–2700 Fe? An indication is in the Materials and Methods sections of the papers by Fittipaldi et al.: “Any aggregate of protein and iron oxides produced during magnetic nanoparticle formation were removed by centrifugation” [18,19]. Just as in the early study of Fischbach and Anderegg [89], which became for many years the source of the misconception that ferritin can take up 4500 Fe, overloading leads to binding of iron on the outside of the protein, and eventually to precipitation [86]. A second problem associated with the Fe(II) loading of ferritin is the competition of the enzymatic oxidation with the background reaction of chemical oxidation by molecular oxygen and/or hydrogen peroxide [90,91]. Oxidation of aqueous Fe^2+^ is strongly dependent on the pH (tenfold increase per pH unit in the range from 6–8); it also increases with temperature and with iron concentration [92,93]; the reaction with H_2_O_2_ is generally even faster [94,95,96]. Fittipaldi et al. carried out the loading of PfF at pH 8.6 and at a temperature of 65 °C. The iron concentration is unknown because the reaction volume was not specified [18,19]. Under these conditions, it is very likely that at least part of the Fe^2+^ was converted to Fe_2_O_3_·xH_2_O before the iron was able to enter the enzyme’s ferroxidase active site. Contrarily, we loaded aerobic PfF at ambient temperature, at pH 7, with small volume additions of anaerobic Fe^2+^ solution, followed by aerobic incubation, plus a final overnight waiting time at 4 °C [87,90]. The procedure was constantly monitored optically at 315 nm, and the extinction coefficient was constant over the whole loading trajectory. Under these conditions, precipitation was never observed. In our hands, the use of H_2_O_2_ as oxidant never led to clean loaded samples (our unpublished observations).

**Figure 4:**
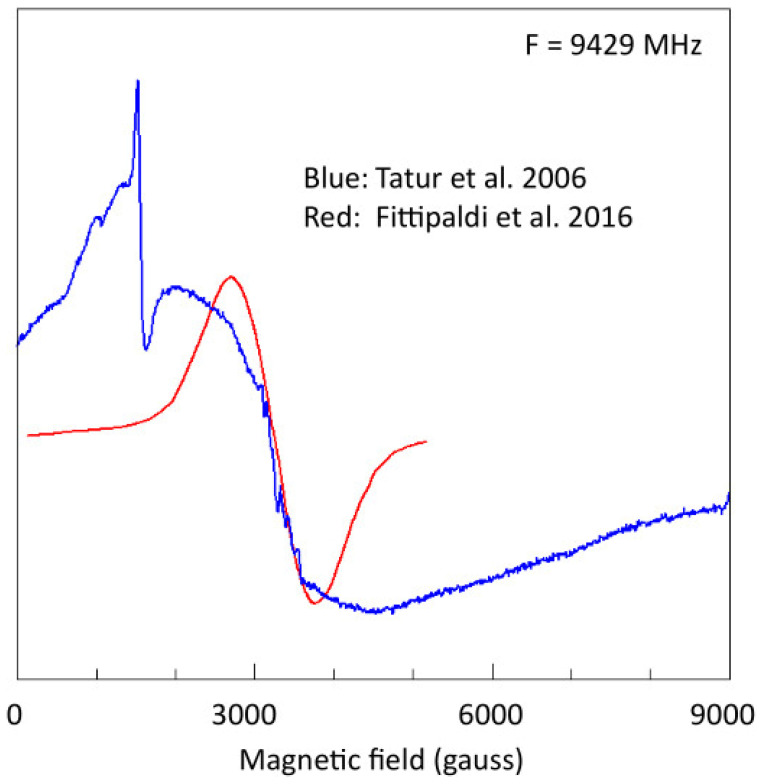
Comparison of EPR spectra from Fe-loaded *Pyrococcus furiosus* ferritin based on two different loading procedures. The blue trace, reported by Tatur et al. [87] for sample *T* = 110 K, is for ferritin loaded with 1140 Fe^2+^ using O_2_ as the oxidant at ambient temperature and pH = 7.0, in a procedure with small stepwise additions and long incubation times [87,90]. The red trace, reported by Fittipaldi et al. [19] for sample *T* = 130 K, and here digitalized from their Figure 1, is for ferritin overloaded with 4500 Fe^2+^ using H_2_O_2_ as the oxidant at a temperature of 65 °C and pH = 8.6 in a titration procedure with constant flow of Fe and H_2_O_2_. The two core signals differ in width by a factor of circa three.

In Figure 4, an EPR spectrum of the core that we published in our original paper on PfF [87] is compared with a spectrum taken at a similar sample temperature by Fittipaldi et al. [19]. The two spectra are quite different in overall width, and the one that we published is similar to spectra published by others for natural (that is, in vivo loaded) mammalian spleen ferritin [13,14,15,97,98]. I conclude that the samples prepared by Fittipaldi et al. are insufficiently biochemically characterized, and, therefore, that the determined magnetic properties do not necessarily apply to loaded PfF.

The giant-spin model has recently also been applied to human liver ferritin by Bossoni et al. [13]. This study differs from those on PfF [18,19] in that a commercial preparation of ferritin was used, that is, no in vivo loading was applied. The preparation was found to contain 1967 ± 78 iron atoms per ferritin [13], that is, well below the circa 3000 Fe maximum [86]. EPR was analyzed in terms of (at least) two different components based on the model that each component is characterized by a single giant-spin value, *S*, and a single *D*-value to describe zero-field interaction, with *S* and *D* related through Equation (12). One can wonder, as is done in the next section, whether such an analysis is meaningful when description of the ZFI is limited to a single parameter only.

## 7. The EPR Spectral-Feature Space of Giant Spins

I have argued, above, that for a system with large spin, say *S* ≈ 10^3^, when the coefficients *B*^q^_k_ of the axial zero-field terms (and presumably also those of different symmetry) of subsequent order are taken to be reduced by, say, four orders of magnitude with each increase in *k*, the ZFI will rapidly diverge towards infinity. Under this condition, one would expect the system to be EPR silent; however, for ferritin cores, this conclusion is contradicted by multiple reports on observable EPR. We are compelled to conclude that order-of-magnitude values for the fourth- and sixth-order ZFI coefficients, reported for SMMs, have no predicting merit for truly giant spins such as in ferritin cores. Combined with (i) the very large number of ZFI terms that would be in principle allowed, (ii) the non-scalability of higher-order ZFI terms in the giant-spin model, (iii) the relatively featureless EPR spectral shapes found for ferritin cores (and similarly for non-biological nanoparticles), (iv) a lack of knowledge on the nature and anisotropy of the EPR line shape, and (v) numerous studies reporting non-homogeneities on morphology and crystallinity, we face the challenge to perform analyses of grossly underdetermined systems. I therefore consider it audacious, but otherwise not well defendable, to draw specific physicochemical conclusions from ferritin core EPR analyses based on a single *D*-value only, such as a temperature-dependent non-isotropic distribution of the easy axis [19], or a two (or more) spin component assignment [13].

**Figure 5:**
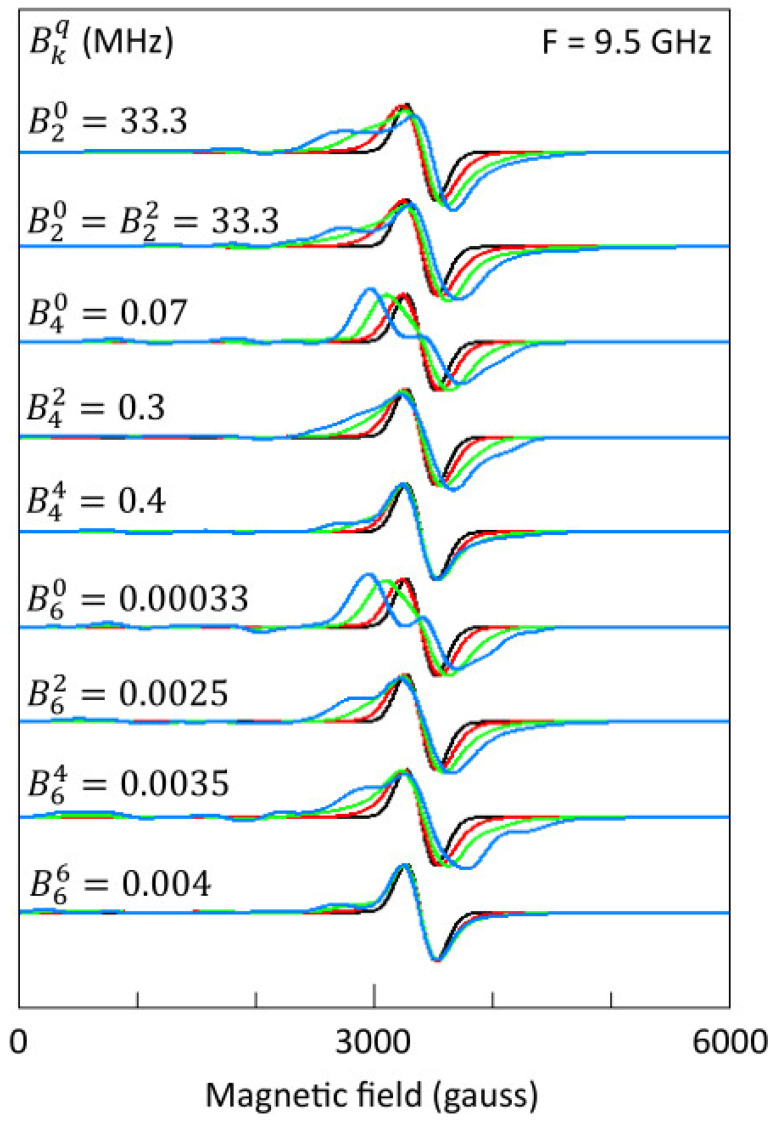
Inventory of line shapes from randomly oriented giant spins that result from switching on individual ZFI coefficients. The orthorhombic-spin Hamiltonian is the sum of Equations (1), (3), and (7), with *g*_iso_ = 2.00 and a Gaussian line shape with FWHM = 300 gauss. Each coefficient *B*^q^_k_ is switched on individually (that is, all other *B*^q^_k_s are set to zero) except for *B*^2^_2_, which is assumed to be equal to *B*^0^_2_ (that is, maximal rhombicity, *E*/*D* = 1/3). The value for *B*^q^_k_, except *B*^2^_2_, is chosen such that induced spectral changes (red traces) are just resolved from the basic spectrum without ZFI (black traces). Subsequently, this value is doubled (green traces) and tripled (blue traces).

A qualitative interpretation of observed EPR spectra is still possible by inventorizing conceivable powder spectral patterns as a function of ZFI coefficients. This approach is explored in Figure 5, in which, for a model spin of *S* = 10, individual second-, fourth-, and sixth-order *B*^q^_k_ coefficients in orthorhombic symmetry are switched on to a level such that spectral changes are starting to become resolved from a model spectrum with given line shape and linewidth (here: Gaussian with FWHM = 300 gauss). Note that, in this approach, interpretation of the magnitude of a coefficient, except for *B*^0^_2_ = *D*/3, is only possible after establishing its non-linear dependence on the system spin in model calculations. For Figure 5 a value of *D* = 100 MHz (=0.0033 cm^−1^) has been assumed, based on approximately similar values quoted for *S*_eq_ = 10 in [13,19]. All |*m*_S_> sublevels are assumed to be equally populated. The base spectrum (black traces) is for zero ZFI with *g*_iso_ = 2. Each *B*^q^_k_ is switched on individually (that is, with all others kept at zero value) except for *B*^2^_2_, which requires a finite value of *B*^0^_2_ based on the inequality *E* ≤ *D*/3 or *B*^2^_2_ ≤ *B*^0^_2_ [99]. In the second set of traces of the figure the maximum value of *E*/*D* = 1/3 has been assumed. Clearly, effects of a finite *E*-value on the powder shape are quite small. The indicated values of the *B*^q^_k_ apply to the red traces; the values are doubled for the green traces and tripled for the blue traces.

Very generally, all terms cause a broadening and subsequently the development of extra structure on both sides of the g value, which is furthermore accompanied by two new features on the low-field side of the base spectrum, one at approximately half field, and one close to zero field. Equivalent fourth- and sixth-order terms cause similar spectral changes, and, therefore, the inclusion of terms of even higher order is not expected to lead to drastically different spectral shapes. For example, *B*^0^_4_ and *B*^0^_6_ each induce a clear splitting of the main line, which is not seen with any of the other terms. Similarly, the terms *B*^4^_4_ and *B*^6^_6_ each create two shoulders, while the main line is hardly affected. Contrarily, *B*^2^_4_ and *B*^2^_6_ each also create two shoulders, but at the same time, the central line is significantly broadened. The term *B*^4^_6_ by itself is interesting in that it causes the strongest low-field extra features and the best resolved shoulder at high field. It is abundantly clear from the effects observed in Figure 5, and from merged effects from any combination of the terms used in Figure 5, perhaps extended with eight- and higher-order terms, that analysis of spectral details using a single *D*-value only will not lead to meaningful physical interpretations.

## 8. Conclusions

The present perspective is intended as a position document: from an EPR spectroscopic perspective, ferritin cores and similar molecular complexes define highly underdetermined systems for quantum-mechanical analysis based on giant-spin Hamiltonians. For ‘similar systems’, one can read not only other proteins with iron storage capacities but also non-biological iron oxide or other nanoparticles. Spectral patterns may be ascribed to single-phase spin systems of random orientation with the aid of inventories of possible patterns as in Figure 5, but at our present state of knowledge, restraint is called for interpreting these patterns in terms of, e.g., species multiplicity or the distribution of physical parameters. Proper in vitro preparation and characterization of ferritin cores in iron-loading experiments of apoferritins is also a matter of concern. Armed with these caveats, the outlook should be towards studies on ferritin cores and other nanoparticles that are better focused by here-discussed theoretical and experimental limitations and possibilities.
